# Electrocardiographic Versus Echocardiographic Left Ventricular Hypertrophy in Severe Aortic Stenosis

**DOI:** 10.3390/jcm10112362

**Published:** 2021-05-27

**Authors:** Aleksandra Budkiewicz, Michał A. Surdacki, Aleksandra Gamrat, Katarzyna Trojanowicz, Andrzej Surdacki, Bernadeta Chyrchel

**Affiliations:** 1 Students’ Scientific Group, Second Department of Cardiology, Jagiellonian University Medical College, 2 Jakubowskiego Street, 30-688 Cracow, Poland; aleksandra.budkiewicz@student.uj.edu.pl (A.B.); msurdacki1997@gmail.com (M.A.S.); aleksandra.gamrat@gmail.com (A.G.); katarzyna.trojanowicz@student.uj.edu.pl (K.T.); 2 Second Department of Cardiology, Institute of Cardiology, Jagiellonian University Medical College, Jakubowskiego Street, 30-688 Cracow, Poland; surdacki.andreas@gmx.net

**Keywords:** electrocardiography, echocardiography, left ventricular hypertrophy, aortic stenosis

## Abstract

Although ECG used to be a traditional method to detect left ventricular hypertrophy (LVH), its importance has decreased over the years and echocardiography has emerged as a routine technique to diagnose LVH. Intriguingly, an independent negative prognostic effect of the “electrical” LVH (i.e., by ECG voltage criteria) beyond echocardiographic LVH was demonstrated both in hypertension and aortic stenosis (AS), the most prevalent heart valve disorder. Our aim was to estimate associations of the ECG-LVH voltage criteria with echocardiographic LVH and indices of AS severity. We retrospectively manually analyzed ECG tracings of 50 patients hospitalized in our center for severe isolated aortic stenosis, including 32 subjects with echocardiographic LVH. The sensitivity of single traditional ECG-LVH criteria in detecting echocardiographic LVH was 9–34% and their respective specificity averaged 78–100%. The ability to predict echocardiographic LVH was higher for S-waves than R-waves (mean area under the receiver operating curve (AUC): 0.62–0.70 vs. 0.58–0.65). Among combinations of R- and S-waves, the discriminating ability was highest for the Cornell voltage (AUC: 0.71) compared to the Sokolow–Lyon, Romhilt and Gubner–Ungerleider voltage (AUC: 0.62–0.68). By multiple regression, peak aortic pressure gradient was positively related to the Sokolow–Lyon (β = 1.7 ± 0.5, *p* = 0.002) and Romhilt voltage (β = 1.3 ± 0.5, *p* = 0.01), but not Cornell (0.5 ± 0.3, *p* = 0.2) or Gubner-Ungerleider voltage (β = 0.0 ± 0.5, *p* > 0.9), regardless of LV mass index. In conclusion, echocardiographic LVH and stenosis severity appear to have distinct associations with traditional ECG-LVH criteria in AS. A moderate diagnostic superiority of the Cornell voltage criterion with regard to anatomic LVH might result from its unique ability to include depolarization vectors in both the frontal and horizontal plane with consequent lesser sensitivity to the confounding effect of obesity.

## 1. Introduction

As early as 50 years ago, left ventricular hypertrophy (LVH) by electrocardiography (ECG) was identified as a predictor of all-cause and cardiovascular (CV) mortality, especially sudden cardiac death, by Framingham Heart Study investigators [1,2,3]. Although ECG used to be a traditional method to detect LVH, its importance has decreased over the years and echocardiography has emerged as a routine technique to diagnose LVH. Admittedly, traditional ECG criteria for LVH, originating from the pre-echocardiographic era, have a relatively low sensitivity (usually not more than 25–40%) against LVH by echocardiography or magnetic resonance imaging [4,5,6,7,8]. However, a low concordance between ECG and ultrasound criteria of LVH cannot be perceived as a simple consequence of the inferiority of ECG as a diagnostic tool compared to modern imaging techniques [8]. Notably, anatomic and electrical LVH appear distinct—albeit partially overlapping—entities providing independent prognostic information, likely linked to different underlying mechanisms [8]. Importantly, “electrical” LVH (i.e., by ECG voltage criteria) is associated with excessive risk of overall mortality, sudden cardiac death and atrial fibrillation irrespective of LV mass and anatomic LVH [8,9,10,11,12]. Thus, an investigation of the associations between echocardiographic LVH and the ECG-LVH voltage criteria is of clinical relevance.

An independent negative prognostic effect of the ECG-LVH beyond echocardiographic LVH was also demonstrated in aortic stenosis (AS) [13], the most prevalent heart valve disorder with LVH, predisposing to heart failure and arrhythmic death. Notably, there is a relative paucity of reports comparing the predictive ability of a wide set of the ECG voltage criteria against anatomic LVH in AS [14]. Our aim was to estimate associations of ECG voltage criteria for LVH with echocardiographic LVH and indices of AS severity.

## 2. Materials and Methods

We retrospectively manually analyzed 12-lead ECG tracings and echocardiographic records from the index hospitalization of patients with isolated severe AS. Severe AS was defined as peak aortic jest velocity ≥ 4 m/s (or mean aortic transvalvular pressure gradient ≥ 40 mmHg) and/or aortic valve area (by continuity equation) < 1.0 cm^2^, in agreement with current recommendations on the echocardiographic assessment of AS [15]. Exclusion criteria included prolonged QRS duration over 120 ms, a history of myocardial infarction, more than mild aortic or mitral regurgitation and significant LV dysfunction (EF < 40%) by echocardiography. In addition, patients with left or right bundle-branch blocks or left anterior fascicular block were also excluded due to distinct criteria for LVH in these conditions [6].

We assessed the relations between echocardiographic LVH (LV mass index >95 g/m^2^ in women and >115 g/m^2^ in men by the Devereux formula from M-mode measurements [16]) and LVH detected by the traditional QRS criteria for LVH. Additionally, the associations were also estimated with LVH defined by LV mass indexed for height (>47 g/m^2.7^ in women and >50 g/m^2.7^ in men) in accordance with the current clinical practice guidelines [17]. The ECG-LVH criteria included R-wave voltage in leads I, aVL, V_5_ and V_6_, as well as the Sokolow–Lyon, Cornell, Romhilt and Gubner–Ungerleider voltage criteria, which combine the amplitudes of R-waves and S-waves [6,17].

The ethics committee of our university approved the protocol as well as the waiver of informed consent to a retrospective data analysis (Approval No.: 1072.6120.260.2020 issued on 24 September 2020).

### Statistical Analysis

Data are presented as mean ± SD or numbers and proportions. Intergroup comparisons were performed by two-tailed Student’s *t*-test and Fisher’s exact test for continuous and categorical variables, respectively. Pearson’s correlation coefficients (r) were computed to test relations between the amplitude of R-waves and S-waves in individual ECG leads.

Sensitivity, specificity, positive and negative predictive values and overall accuracy were calculated to estimate the diagnostic ability of the ECG-LVH criteria to detect echocardiographic LVH. Then, Cohen’s kappa was computed as a measure of concordance between ECG and echocardiography in terms of LVH beyond that which would be expected by chance alone [18]. McNemar’s test was used to estimate a systematic difference between the results obtained by ECG and echocardiography with regard to LVH [16]. Additionally, the receiver operating characteristic (ROC) curve analysis was applied to assess the predictive ability of the Sokolow–Lyon, Cornell, Romhilt and Gubner–Ungerleider voltage, as well as that of the amplitude of R-waves and S-waves in individual ECG leads across all possible threshold values of the respective voltage as a continuous predictor.

Multiple regression was used to identify predictors of Sokolow–Lyon, Cornell, Romhilt and Gubner–Ungerleider voltage, including LV mass indexed to body-surface area, peak aortic pressure gradient, a marker of AS severity, and age and body mass index as independent variables. To adjust for the confounding effect of obesity on LVH definition [19], these associations were also estimated with LV mass indexed for height^2.7^. Mean non-standardized regression coefficients (β) and their standard errors (SEM) were reported, corresponding to the change of each voltage associated with a given increment of the predictor variable.

A *p*-value < 0.05 was assumed significant, except for correlation coefficients when adjusted *p*-value < 0.0015 was inferred significant due to Bonferroni’s correction for multiple comparisons (n = 32).

All calculations were carried out by the Statistica 64 (data analysis software system, version 13.3.704.0 (TIBCO Software Inc. (2017), Palo Alto, CA, USA).

## 3. Results

Out of 83 pre-screened subjects with complete data, 50 patients entered the final analysis (mean age: 77 ± 10 years; 30 women and 20 men), including 32 subjects with echocardiographic LVH. Clinical and echocardiographic characteristics according to echocardiographic LVH are shown in Table 1.

The sensitivity of single traditional ECG-LVH criteria in detecting echocardiographic LVH was 9–34% and their respective specificity averaged 78–100%; the Cornell voltage criterion exhibited the highest sensitivity (34%) and the lowest specificity (78%) (Table 2). The presence of any of the traditional ECG-LVH criteria improved sensitivity (66% vs. 9–34%), negative predictive value (48% vs. 36–41%), the overall accuracy (62% vs. 38–50%) and agreement with echocardiographic LVH (Cohen’s kappa: 0.20 vs. −0.01–0.14; McNemar’s test: vs. *p* = 0.6 vs. *p* < 0.001–*p* = 0.0014), albeit at the cost of decreased specificity (56% vs. 78–100%). Nevertheless, a Cohen’s kappa value of 0.0.20 indicated only a slight agreement between echocardiographic LVH and the presence of any of the above set of traditional ECG-LVH criteria (Table 2).

**Figure 1:**
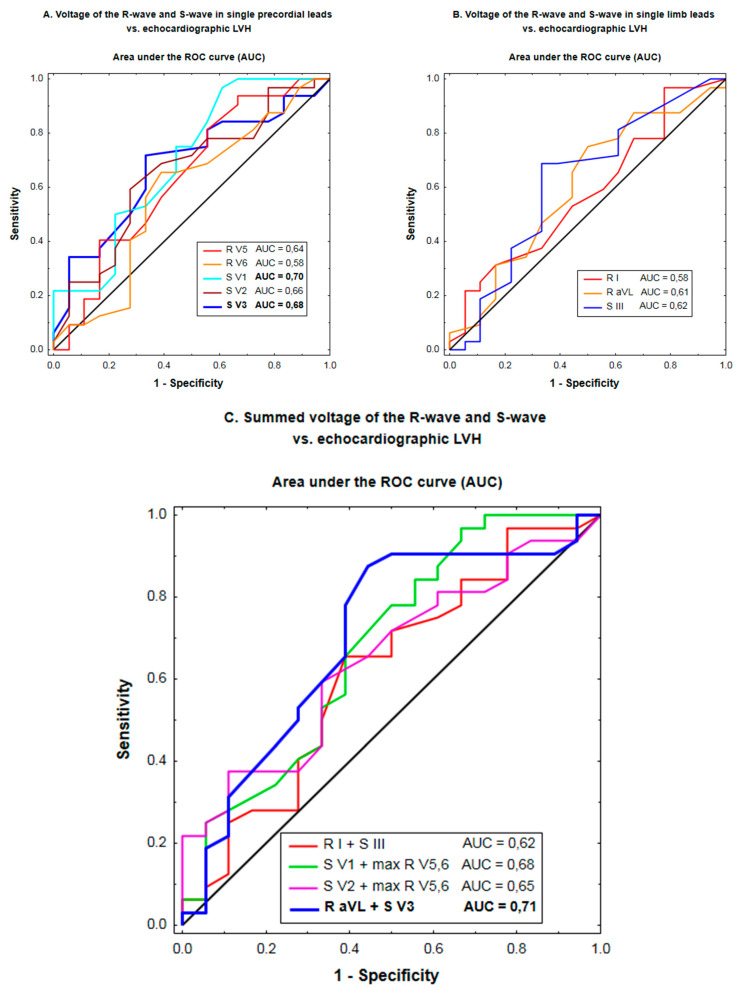
Discriminating ability of the voltage of the R-wave and S-wave in single precordial leads (**A**), single limb leads (**B**), and combinations of the amplitude of R-waves and S-waves (**C**) for the prediction of echocardiographic LVH according to the receiver operating characteristic (ROC) curve analysis. *p*-values below 0.05 are denoted in bold.

According to the ROC analysis, the discriminating ability was higher for S-waves than R-waves (mean AUC: 0.62–0.70 vs. 0.58–0.65) (Table 3, Figure 1A,B). Among combinations of R- and S-waves, the discriminating ability was highest for the Cornell voltage compared to the Sokolow–Lyon, Romhilt and Gubner–Ungerleider voltage (mean AUC: 0.71 vs. 0.62–0.68) (Table 3, Figure 1C).

The definition of LVH by height-indexed LV mass did not substantially change the results.

The amplitudes of R-waves in leads I and aVL, R-waves in leads V_5_–V_6_, and S-waves in leads V_1_–V_2_, were strongly and significantly interrelated within each of the above groups of ECG leads (RI vs. RaVL, r = 0.83, *p* < 0.001; RV_5_ vs. RV_6_: r = 0.81, *p* < 0.001; SV_1_ vs. SV_2_: r = 0.60, *p* < 0.001). In contrast, there were either no or only very weak positive correlations between the amplitudes of R- and S-waves in the limb leads on the one part and their counterparts in the precordial leads on the other part (RI and RaVL vs. RV_5_–V_6_: r = −0.22–0.15, *p* > 0.12; SIII vs. SV_1_–V_3_: r = −0.04–0.18, *p* > 0.2).

By multiple regression, the relationship with LV mass indexed to body-surface area was most significant for the Cornell voltage (β ± SEM: 0.7 ± 0.2, *p* = 0.005) upon adjustment for age, body mass index and peak aortic pressure gradient (Table 4). In contrast, peak aortic gradient was positively related to the Sokolow–Lyon (β = 1.7 ± 0.5, *p* = 0.002) and Romhilt voltage (β = 1.3 ± 0.5, *p* = 0.01) but not Cornell (β = 0.5 ± 0.3, *p* = 0.2) or Gubner-Ungerleider voltage (β = 0.0 ± 0.5, *p* > 0.9) (Table 4). These associations were very similar when LV mass was indexed for height^2.7^ (Table 5).

## 4. Discussion

Our salient finding was that echocardiographic LVH and AS severity had distinct associations with traditional ECG criteria for LVH.

### 4.1. The ECG-LVH Criteria vs. Echocardiographic LVH

We found a higher discriminating ability of the amplitude of S-waves compared to R-waves with regard to echocardiographic LVH. This observation can be linked to a better representation of abnormal wave-front propagation in LVH by the latter part of the QRS complex, corresponding to the S-wave and reflecting the depolarization of LV midwall and epicardial fibers and basal LV infero-lateral segments, as proposed by Peguero et al. [20] for precordial S-waves in leads V_3_ and V_4_.

Additionally, we also observed a better diagnostic ability of the Cornell voltage in comparison to other criteria combining R-wave and S-wave amplitudes, i.e., the Sokolow–Lyon, Romhilt and Gubner–Ungerleider voltage. The moderate superiority of the Cornell voltage might result from a unique ability of the former to include depolarization vectors in both the frontal (RaVL) and horizontal plane (SV_3_). In contrast, the Sokolow–Lyon, Romhilt and Gubner–Ungerleider voltage reflect QRS amplitude either in the horizontal or frontal plane only, corresponding to the precordial and limb ECG leads, respectively. This might decrease limit their diagnostic ability because elevations of the QRS amplitude in precordial and limb leads may be independent of each other for geometric reasons. In agreement with this concept, the amplitudes of R- or S-waves in precordial leads (RV_5_, RV_6_ or SV_1_–V_3_) were either not related or only very weakly positively correlated to their counterparts in limb leads (RI, RaVL or SIII).

The highest sensitivity of the Cornell voltage criterion for echocardiographic LVH (34%) is consistent with previous studies of subjects with moderate-to-severe AS by Sjöberg et al. [21] and Bula et al. [14] (50% and 38%, respectively), while the sensitivity of the Sokolow–Lyon criterion was similar in those studies (29%) and in the present study (28%). In addition, since concentric LVH was a predominant form of LVH in our AS patients, a better discriminating performance of the Cornell voltage criterion for LVH prediction might be related to its higher sensitivity in concentric versus eccentric LVH (16.5% vs. 11.1%, respectively), as shown by Oikonomou et al. [22] in 1592 participants of the population-based Corinthia study. Additionally, already over 20 years ago, Tomita et al. [23] reported higher Cornell and Sokolow–Lyon voltage in concentric than eccentric LVH.

Second, as the majority of our study subjects were overweight, our findings appear consistent with the results by Okin et al. [24] who reported an enhanced superiority of the Cornell over Sokolow–Lyon voltage in overweight subjects. Admittedly, we observed only insignificant tendencies of lower Romhilt and Sokolow–Lyon voltage at higher body mass index by multiple regression, in contrast to significant respective associations in the study of Bula et al. [14], which may be related to a lower number of AS subjects in our study group. Nonetheless, the confounding effect of obesity on the performance of the ECG-LVH criteria was most pronounced for the Sokolow–Lyon voltage [24,25,26]. In addition, leftward QRS axis deviation, frequently accompanying obesity, was shown to decrease the Sokolow–Lyon, but not Cornell voltage [26].

Third, in a computer simulation study by Bacharova et al. [27], the impaired electrical intercellular coupling, known to coexist with LVH, was shown to decrease the QRS voltage in all ECG leads except aVL, a component of the Cornell voltage, which could also facilitate the predictive ability of the Cornell voltage.

### 4.2. The ECG-LVH Criteria vs. Aortic Stenosis Severity

In addition to the above findings, linked presumably to altered myocardial excitation wave-front propagation in LVH, we also observed associations of LVH criteria and peak aortic pressure gradient, an estimate of stenosis severity, which remained significant upon adjustment for LV mass index, age and body mass index. This observation is consistent with an independent relationship between electrical LVH and hemodynamic characteristics, reported in previous studies of AS subjects [14,28]. In particular, we found positive associations of the Sokolow–Lyon voltage index and Romhilt voltage, but not Gubner–Ungerleider or Cornell voltage with maximal aortic pressure gradient, which is concordant with the respective findings of Bula et al. [14], who recently described analogous relations with peak aortic jet velocity. Likewise, Greve et al. [28] described significantly higher peak aortic velocity in asymptomatic AS subjects with ECG-LVH by the Sokolow–Lyon voltage index; nevertheless, beyond that index exclusively Cornell voltage-duration product, not Cornell voltage, were estimated in their study.

It may be speculated that the association of a higher peak aortic pressure gradient and the Sokolow–Lyon voltage might contribute to a negative prognostic value of the latter irrespective of increased LV mass via augmented LV pressure overload in AS [13]. It is noteworthy that the Sokolow–Lyon voltage criterion independently predicted future adverse CV events over about 4 years regardless of LV mass, stenosis severity and clinical characteristics in 1518 asymptomatic patients with mild or moderate AS at baseline, participating in the SEAS study [13]. In particular, the adjusted risk of heart failure hospitalization attributable to AS was three to fourfold higher in 260 patients who met the Sokolow–Lyon voltage criterion compared to their 1258 counterparts without ECG-LVH [13].

Additionally, subclinical LV dysfunction could also affect prognosis in subjects with ECG-LVH. In support of this concept, in patients with hypertension, the Sokolow–Lyon voltage and/or the Cornell voltage or product were related to the degree of subclinical LV systolic dysfunction by reduced global longitudinal strain and increased inner-to-outer ratio of circumferential strain, as well as diastolic dysfunction by tissue doppler imaging [29]. Moreover, the positive association between the Sokolow–Lyon voltage and reduced LV longitudinal strain was also observed in severe AS, which was maintained upon multivariate adjustment including LV mass index [30].

Therefore, it can be proposed that independent relations between some ECG-LVH criteria and longitudinal LV systolic dysfunction [29,30], a powerful mortality predictor in AS despite preserved EF [31,32], could contribute to the ability of the Sokolow–Lyon voltage to predict heart failure hospitalization even after multivariate adjustment, including a correction for LV mass in AS [13]. Additionally, longitudinal LV dysfunction is paralleled by interstitial collagen deposition [32,33,34], which may apparently increase LV mass without augmented QRS voltage, thereby contributing to a poor agreement of echocardiography and ECG in terms of LVH. Nevertheless, these considerations remain speculative as our study was based on a retrospective cross-sectional analysis of in-hospital medical records, so that novel echocardiographic techniques were unavailable in our study group.

### 4.3. Study Limitations

First, the study was limited by a retrospective study design and low number of study subjects. However, we analyzed only medical records of patients with isolated severe AS free of His bundle branch blocks and intraventricular conduction defects, and without significant LV dysfunction or a history of myocardial infarction. Second, we estimated anatomic LVH by echocardiography, while magnetic resonance imaging provides more reliable results. Third, medical treatment was not uniform in the study subjects; nevertheless, there were only minor differences in medication use between patients with and without LVH.

## 5. Conclusions

Echocardiographic LVH and stenosis severity appear to have distinct associations with traditional ECG-LVH criteria in AS. A higher discriminating ability of the amplitude of S-waves versus R-waves with regard to echocardiographic LVH can be linked to a better representation of abnormal depolarization wave-front propagation in LVH by the latter part of the QRS complex, corresponding to the S-wave. A moderate diagnostic superiority of the Cornell voltage criterion might result from its unique ability to include depolarization vectors in both the frontal and horizontal plane with consequent lesser sensitivity to the confounding effect of obesity. Independent associations of AS severity with the Sokolow–Lyon and Romhilt voltagecould hypothetically contribute to a negative prognostic effect of ECG-LVH beyond anatomic LVH in AS.

## Figures and Tables

**Table 1:** Characteristics of patients according to echocardiographic LVH.

Characteristic	Echocardiographic LVH n = 32	No Echocardiographic LVH n = 18	*p*-Value
Age, years	77 ± 10	77 ± 11	NS
Women/men, n	19/13	11/7	NS
Hypertension, n (%)	31 (97%)	15 (83%)	NS
Diabetes, n (%)	16 (50%)	10 (56%)	NS
Body mass index, kg/m^2^	27.8 ± 4.2	25.2 ± 3.7	0.03
eGFR, mL/min/1.73 m^2^	67 ± 16	74 ± 16	NS
LV mass index, g/m^2^	141 ± 34	86 ± 14	<0.001
LV end-diastolic diameter, mm	48 ± 7	42 ± 5	0.0015
Relative LV wall thickness	0.57 ± 14	0.50 ± 0.10	0.10
Concentric LV geometry (relative LV wall thickness ≥0.42)	29 (90%)	14 (78%)	NS
LV ejection fraction, %	59 ± 9	60 ± 9	NS
Peak aortic gradient, mm Hg	91 ± 25	74 ± 26	0.03
Mean aortic gradient, mm Hg	58 ± 17	45 ± 19	0.02
Aortic valve area, cm^2^	0.7 ± 0.2	0.8 ± 0.2	0.03
Medication, n (%)			
ACEI or ARB	26 (81%)	15 (83%)	NS
Beta-blockers	24 (75%)	11 (61%)	NS
Diuretics	21 (66%)	10 (56%)	NS
Calcium-channel blockers	11 (34%)	6 (33%)	NS

Data are presented as mean ± standard deviation or numbers (percentages). Abbreviations: ACEI, angiotensin-converting enzyme inhibitor; ARB, angiotensin receptor blocker; CCB, calcium-channel blockers; eGFR, estimated glomerular filtration rate by the CKD-EPI formula; LV, left ventricular; LVH, left ventricular hypertrophy; NS, non-significant.

**Table 2:** Association of ECG criteria for LVH with echocardiographic LVH.

ECG Criteria for LVH	Sensitivity	Specificity	Positive Predictive Value	Negative Predictive Value	Accuracy	Cohen’s Kappa ^a^	McNemar’s Test ^b^
**Single criterion**							
Max. RV_5,6_ > 2.6 mV	9%	89%	60%	36%	38%	−0.01	<0.001
RV_6_ > RV_5_	13%	94%	80%	38%	42%	0.05	<0.001
SV_1_ + max RV_5,6_ > 3.5 mV	28%	89%	82%	41%	50%	0.14	<0.001
SV_2_ + max RV_5,6_ > 4.5 mV	16%	100%	100%	40%	46%	0.12	<0.001
RI > 1.5 mV	22%	94%	88%	41%	48%	0.13	<0.001
RI + SIII > 2.5 mV	22%	89%	78%	39%	46%	0.08	<0.001
RaVL ≥ 1.1 mV	31%	83%	77%	41%	50%	0.12	<0.001
RaVL + SV_3_ > 2.0 mV (W) RaVL + SV_3_ > 2.8 mV (M)	34%	78%	73%	40%	50%	0.10	0.0014
**Any of the above criteria**	66%	56%	72%	48%	62%	0.20	0.6

^a^ an estimate of concordance between ECG and echocardiography with regard to LVH beyond that which would be expected by chance alone. ^b^ *p* < 0.05 indicates a lack of agreement comparing the ECG-LVH criteria against echocardiographic LVH due to a systematic difference between the results obtained by ECG and echocardiography with regard to LVH. Abbreviations: M, men; W, women; other abbreviations as in Table 1.

**Table 3:** Comparison of the discriminating ability of the voltages of the R-wave and S-wave in individual ECG leads for prediction of echocardiographic LVH across all possible threshold values of the voltage as a continuous predictor.

QRS Voltage	Area under the Receiver Operating Characteristic (ROC) Curve Mean (95% Confidence Interval)	*p*-Value ^a^
**R-waves**		
RV_5_	0.64 (0.47–0.81)	0.1
RV_6_	0.58 (0.41–0.76)	0.4
Max RV_5,6_	0.65 (0.48–0.82)	0.08
RI	0.58 (0.41–0.75)	0.4
RaVL	0.61 (0.44–0.77)	0.2
**S-waves**		
SIII	0.62 (0.45–0.79)	0.2
SV_1_	0.70 (0.54–0.86)	**0.015**
SV_2_	0.66 (0.50–0.82)	0.06
SV_3_	0. 68 (0.53–0.83)	**0.02**
**Combinations of R-waves and S-waves**		
SV_1_ + max RV_5,6_	0.68 (0.52–0.84)	**0.03**
SV_2_ + max RV_5,6_	0.65 (0.49–0.80)	0.06
RI + SIII	0.62 (0.45–0.78)	0.2
RaVL + SV_3_	0.71 (0.55–0.86)	**0.01**

^a^ *p*-values below 0.05 are denoted in bold.

**Table 4:** Multiple regression analysis of predictors associated with the Cornell, Sokolow–Lyon, Romhilt and Gubner–Ungerleider voltage criteria for LVH with LV mass indexed to body-surface area.

Predictors of the Cornell Voltage (RaVL + SV_3_)	Non-Standardized Regression Coefficient (mean ± SEM)	*p*-Value ^a^
LV mass index, per increment by 10 g/m^2^	0.7 ± 0.2	**0.005**
Peak aortic pressure gradient, per rise by 10 mmHg	0.5 ± 0.3	0.2
Age, per 10-year increase	−2.0 ± 0.9	**0.03**
Body mass index, per rise by 2.5 kg/m^2^	0.6 ± 0.5	0.24
**Predictors of the Sokolow–Lyon voltage** **(SV_1_ + max RV_5,6_)**		
LV mass index, per increment by 10 g/m^2^	0.7 ± 0.4	0.06
Peak aortic pressure gradient, per rise by 10 mmHg	1.7 ± 0.5	**0.002**
Age, per 10-year increase	0.7 ± 1.4	0.6
Body mass index, per rise by 2.5 kg/m^2^	−0.7 ± 0.8	0.4
**Predictors of the Romhilt voltage** **(SV_2_ + max RV_5,6_)**		
LV mass index, per increment by 10 g/m^2^	0.9 ± 0.4	**0.02**
Peak aortic pressure gradient, per rise by 10 mmHg	1.3 ± 0.5	**0.01**
Age, per 10-year increase	−0.5 ± 1.4	0.7
Body mass index, per rise by 2.5 kg/m^2^	−1.0 ± 0.8	0.2
**Predictors of the Gubner–Ungerleider voltage** **(RI + SIII)**		
LV mass index, per increment by 10 g/m^2^	0.6 ± 0.4	0.08
Peak aortic pressure gradient, per rise by 10 mmHg	0.0 ± 0.5	>0.9
Age, per 10-year increase	−1.9 ± 1.4	0.2
Body mass index, per rise by 2.5 kg/m^2^	1.0 ± 0.8	0.2

^a^ *p*-values below 0.05 are denoted in bold. Abbreviations: SEM, standard error of the mean; other abbreviations as in Table 1.

**Table 5:** Multiple regression analysis of predictors associated with the Cornell, Sokolow–Lyon, Romhilt and Gubner–Ungerleider voltage criteria for LVH with LV mass indexed to height^2.7^.

Predictors of the Cornell Voltage (RaVL + SV_3_)	Non-Standardized Regression Coefficient (Mean ± SEM)	*p*-Value ^a^
LV mass index, per increment by 5 g/m^2.7^	0.8 ± 0.3	**0.004**
Peak aortic pressure gradient, per rise by 10 mmHg	0.4 ± 0.3	0.2
Age, per 10-year increase	−2.0 ± 0.9	**0.03**
Body mass index, per rise by 2.5 kg/m^2^	0.3 ± 0.6	0.6
**Predictors of the Sokolow–Lyon voltage** **(SV_1_ + max RV_5,6_)**		
LV mass index, per increment by 5 g/m^2.7^	0.7 ± 0.4	0.08
Peak aortic pressure gradient, per rise by 10 mmHg	1.7 ± 0.5	**0.002**
Age, per 10-year increase	0.7 ± 1.4	0.6
Body mass index, per rise by 2.5 kg/m^2^	−1.0 ± 0.9	0.3
**Predictors of the Romhilt voltage** **(SV_2_ + max RV_5,6_)**		
LV mass index, per increment by 5 g/m^2.7^	0.8 ± 0.4	**0.04**
Peak aortic pressure gradient, per rise by 10 mmHg	1.3 ± 0.5	**0.01**
Age, per 10-year increase	−0.5 ± 1.4	0.7
Body mass index, per rise by 2.5 kg/m^2^	−1.2 ± 0.9	0.16
**Predictors of the Gubner–Ungerleider voltage** **(RI + SIII)**		
LV mass index, per increment by 5 g/m^2.7^	0.9 ± 0.4	**0.02**
Peak aortic pressure gradient, per rise by 10 mmHg	0.1 ± 0.5	0.8
Age, per 10-year increase	−2.0 ± 1.3	0.13
Body mass index, per rise by 2.5 kg/m^2^	0.6 ± 0.8	0.5

^a^ *p*-values below 0.05 are denoted in bold. Abbreviations as in Table 1 and Table 4.

## Data Availability

The data presented in this study are available on request from the corresponding author.
